# Evolutionary Pattern of Asian HIV-1 Subtype B from 1990 to 2007: *In Silico* Analysis Based on Envelop Protein

**DOI:** 10.1100/2012/978917

**Published:** 2012-05-03

**Authors:** Sobia Kanwal, Tariq Mahmood

**Affiliations:** Faculty of Biological Sciences, Quaid-i-Azam University, Islamabad 45320, Pakistan

## Abstract

HIV-1 envelop gene is a major target for vaccine development. Envelop protein and its V3 loop is shown to be important determinant of HIV-1 pathogenecity. Herein, the evolutionary pattern of most prevalent HIV-1 subtype B in Asia is determined by analyzing envelop protein and V3 domain based on the 40 randomly selected sequences of HIV-1 from database (Los Alamos), divided into four groups since 1990–2007. Construction of envelop protein phylogeny by using MEGA 5 exhibit the active mutation pattern, increase in potential N-glycosylation sites which were predicted by using online software SignalP-NN. An online available tool Drawgram was used for multiple sequence alignment (MSA) of HIV-1 subtype B envelop region and V3 loop while the alignment was rechecked by using CLUSTAL W and further was analyzed for GPGX motif and conserved region in V3 loop. Variation at fourth position of the GPGX motif and 60% conservation was found in V3 loop. Hence, this diversifying pattern of envelop protein in the Asia formulates the HIV-1 strains more pathogenic during the period of 17 years. These findings might help in understanding significant structural and functional constrains of the mutant viral strains and ultimately in vaccine development.

## 1. Introduction

High genetic variability of HIV-1 (Human immunodeficiency virus I) is characterized and classified into three distinctive groups; group M (main), group O (outliner), and group N (non-M/non-O) [1–3]. Majority of the global epidemic of the infection is caused by group M which may be further subdivided into nine nonrecombinant subtypes (A-D, F-H, J, and K) and at least 34 intersubtypes recombinant forms, termed as circulating recombinant forms (CRF) [4] distributed on the basis of geographical locations. Subtype B is circulating in United States and Europe, in addition, two genetic forms of subtype B and CRF01-AE are dominant in South East Asia while subtype A is most prevalent in European countries [5].

Envelop gene plays an important role in entry and fusion of HIV-I and is a major target for vaccine development studies. Envelop protein gp160 is encoded by envelop gene involved in host cell-HIV interaction. It is composed of two noncovalently bound subunits, the external glycoprotein gp120 and the transmembrane gp41. External glycoprotein gp120 plays an important role in entry of the virus into the cell by binding to the specific receptors on the surface of the target cell. These receptors are CD4 and chemokine receptors known as HIV-1 coreceptors [6]. The chemokine receptors CXCR4 and CCR5 are the two major coreceptors used by HIV-I isolates [7]. The binding of the chemokine receptor requires the presence of selected amino acids in gp120 (specifically within the V3 loop, and also in other regions), providing greater affinity to CCR5 or CXCR4, and therefore, the viral tropism [8–11]. The gp120 portion of envelop has been generally classified into five hypervariable regions (V1 to V5) with conserved regions interspersed [12]. Among these variable regions, the V3 is one of the most important determinants of viral tropism and coreceptor usage [13], as it contains major antigenic and neutralizing epitopes in HIV-1 which are well exposed upon CD4-binding [14]. This third variable region (V3 loop) of the HIV-1 gp120 also plays an important role in the mechanism controlling the fusion between envelop of HIV-I and the cell membrane of the target cells [15]. Hence, it is considered as the fusion domain whose sole function would be to initiate the fusion process after an initial interaction between gp120 and CD4 on the surface of the CD4+ cells [7]. 

Further, gp120 is also extensively glycosylated protein, containing 24–26 potential glycosylation sites (24 for HIV-INL4-3 strain, 26 for HIV-ISF2) [16]. Glycosylation of envelop gene is crucial in all phases of HIV biology that track from proper envelop folding, processing of virus transmission, and the immune evasive mechanisms [17–19]. Therefore, the systematic analysis of the glycosylation profiles of envelop could provide precious insights in designing well envelop (env) immunogens. A decreased number of N-linked glycosylation sites in gp120, especially within and around the V3 region, have been demonstrated during evolution from R5 to the X4 phenotype [20, 21]. 

Several amino acids residues are extremely conserved in V3 loop due to their functional importance among HIV-1 variants. However, the vast majority of the HIV-1 sequences, and their putative common SIVcpz ancestors, have the GPGX signature pattern (motif) at the central portion of V3 loop of the envelop gene [22]. This conserved motif in a variable region exhibits purifying selection pressure. Some lineages, however, have been found worldwide with alternate signature patterns, such as the GWGR subtype B variant in which the proline residue is substituted by a tryptophan [23, 24]. This variant was first recorded in Japan in early 1990s [25] but a serologic study suggests its presence in Brazil since 1983 [17]. 

The present study is the first attempt to track the evolutionary pattern of HIV-1 in Asia over the period of 17 years. The biological activity and pathogenicity of the most prevalent HIV-1 subtype B in Asia was determined based on the available sequences of HIV-1 in database (Los Alamos) during the time frame of 1990–2007. 

## 2. Material and Methods

Evolutionary pattern of HIV-1 subtype B was tracked by analyzing the envelop protein and its V3 domain.

### 2.1. Dataset

The sequences used in the study were divided into four groups, that is, each group constitutes the sequences from four years:

Group I: 1990–1994,
Group II: 1995–1999,
Group III: 2000–2004,
Group IV: 2005–2007.

In total, 10 sequences of HIV-1 subtype B from each group were obtained from Los Alamos HIV database (http://hiv-web.lanl.gov/). Two type of analysis were performed based on envelop protein and V3 domain of the downloaded sequences.

### 2.2. Phylogenetic Analysis Based on Envelop Protein

Forty sequences of envelop protein were randomly selected and aligned using CLUSTALW (parameters reading DNA Pairwise Parameters; Gap opening penalty: 10, Gap extension penalty: 0.1, Multiple Parameters; Gap opening penalty: 10, Gap extension penalty: 0.2, protein weight matrix: BLOSUM, Residue specific penalties: ON, Hydrophilic penalties: ON, Gap separation distance: 4, End gap separation: OFF, negative matrix OFF, Delay divergent cutoff: 30%). To obtain phylogenetic tree alignment was analyzed in MEGA 4 tool (parameters reading; Tree inference Method: neighbor joining, Include sites: Gaps/missing data: complete deletion, substitution model: Amino p-distance, Substitutions to include: All). 

### 2.3. Prediction of N-Glycosylation Sites in Envelop Protein

N-linked glycosylation sites of HIV-1 envelop protein were predicted by using online software SignalP-NN.

### 2.4. Alignment of V3 Domain

ClustalW was used for the alignment of V3 motifs to check the level of conservation of this region. The conserved region was calculated manually.

### 2.5. Analysis of GPGX Motif

HIV-1 envelop protein sequences were aligned by using ClustalW of online software Drawgram to find out conserved GPGX motif.

### 2.6. Determination of Conserved Region

Used Sequences were aligned through multiple sequence alignment (MSA) of ClustalW from online software Drawgram.

## 3. Results and Discussion

### 3.1. Phylogenetic Profile of Envelop Protein

**Figure 1:**
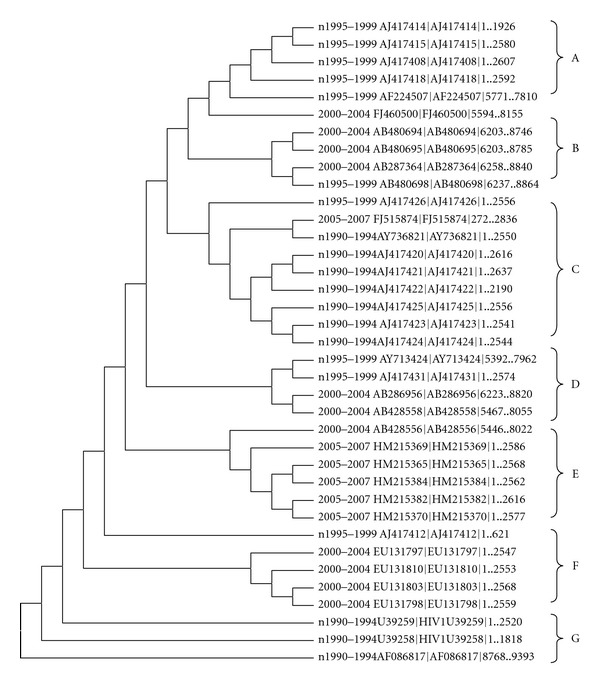
Neighbour-joining phylogenetic tree (constructed using MEGA 5) based on amino acid sequences of envelop protein from HIV-1 subtype B in Asia.

Phylogenetic analysis of the envelop protein sequences in Asian strains of subtype B was performed (Figure 1). The phylogenetic tree representing the sequence relationships by amino acid substitutions identified distinct genetic subgroups exhibiting that viruses within these lineages were genetically closely related. 

Isolates from 1990s make the root of the tree and also exhibits the early evolution of the isolates. Strains from groups year 2000 to 2004 and from 2005 to 2007 clustered together and strains from 1990s act as their root. However, strains from 1990 to 1994 make separate cluster. The clustering did not correlate closely with chronological origin of the sequences. It was observed from the clustering pattern that strains from 1990–1995 remained resistant to selection pressure and act as the root of the tree, while strains from other groups exhibit the high mutation rate demonstrated by their branching pattern. The differences in the evolutionary characteristics found could be attributed to a different selection pattern of the strains by the nature.

Consequently, high mutation rate and divergence have been shown by almost all groups while some viral lineages have shown extensive branching pattern which may be due to negative selection of the strains by the nature (Figure 1, Clade B, C, D, and E). An increase in envelope sequence heterogeneity has also been observed in an infected population over the period of time [8, 9]. This increase in sequence heterogeneity may help in viral escape from host immune response. Other clades of the tree exhibit the strong selection and remain less divergent over the period of time (Figure 1, Clade A and F). The viral robustness is a key factor affecting the viral virulence which measures the capacity of a virus to cause disease [22]. Genetic analysis of envelop gene, particularly the third variable domain (V3), showed that the interhost (intrasubtype) variation increased over the course of the AIDS epidemic [26].

### 3.2. Number of Predicted N-linked Glycosylation Sites

**Figure 2:**
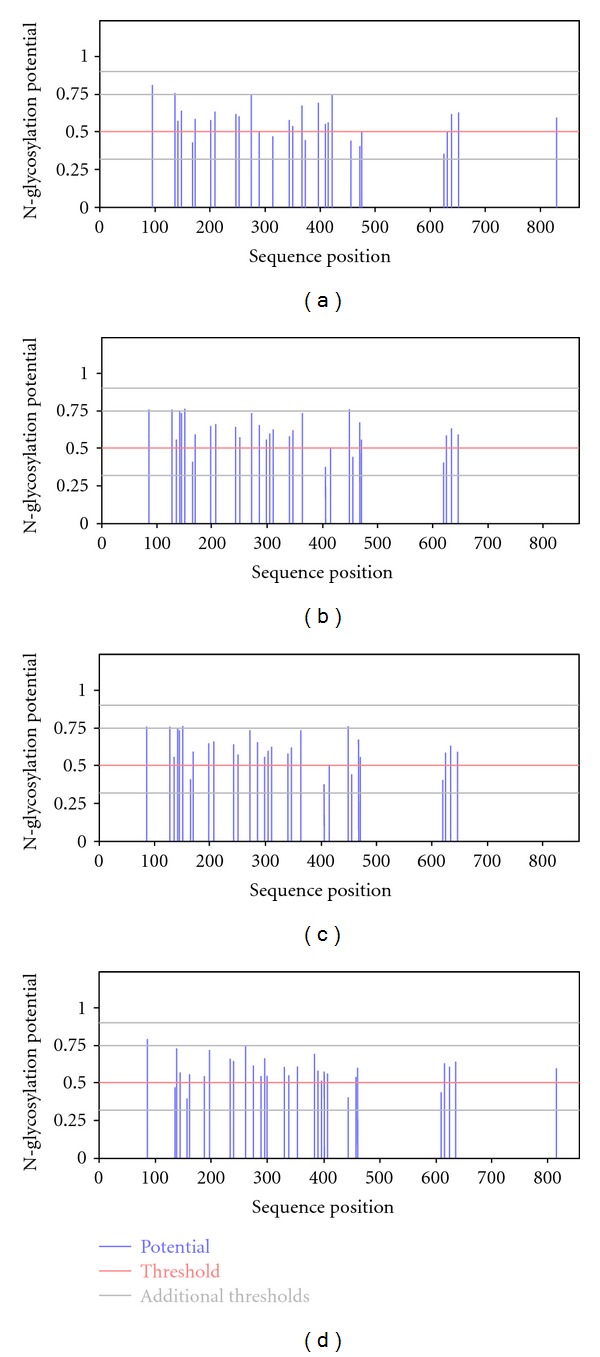
Potential N-glycosylation sites predicted from SignalP-NN. (a) Group I (1990–1994). (b) Group II (1995–1999). (c) Group III (2000–2004). (d) Group IV (2005–2007).

Increase in N-linked glycosylation sites was observed through the period of 17 years from 1990 to 2007. This might serve to protect viruses from neutralizing antibodies. Glycosylation by the host cell can vigorously maneuver the folding, stability, and biological function of virus-encoded proteins [27, 28]. The predicted glycosylation site calculated by using the software SignalP-NN in the period of 1990–1994 is 20, from 1995–1999 is 25, from 2000–2004 is 26, and from 2005–2007 is 30 (Figure 2). 

The N-linked glycosylation (NLG) of viral envelop proteins, through the formation of a "glycan shield," is one of the major mechanisms for inhibiting or minimizing virus neutralizing antibody response [29] which promotes viral persistence and immune evasion. Further, the N-linked glycosylation pattern has also been shown to have a varied effect on the stimulation of neutralizing antibodies in HIV-1 [30, 31]. Consequently, alterations in glycosylation events influencing co-receptor utilization will also allow the virus to escape the controlling responses of the CC chemokines *in vivo*, which are believed to play an effective role in inhibiting viral replication and slowing disease progression [32]. 

### 3.3. Analysis of V3 Domain

**Figure 3:**
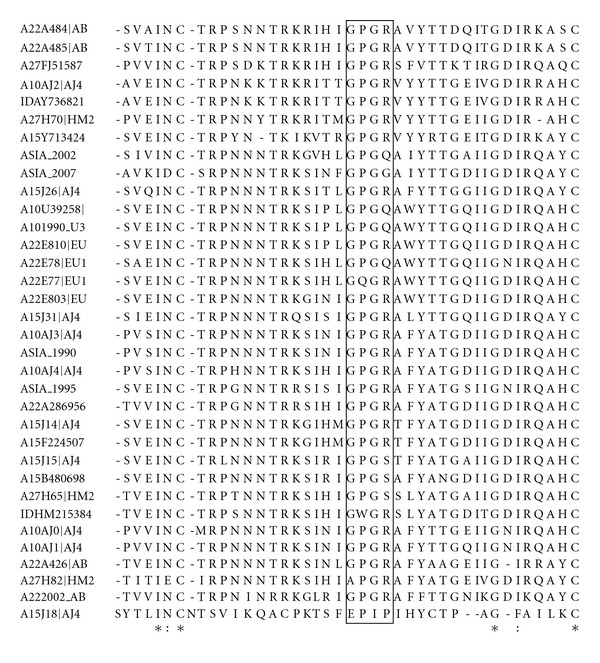
Multiple sequence alignment showing the variation a GPG**X** motif.

Multiple sequence alignment (MSA) describes the variation at fourth position of the tetrapeptide GPGX (Figure 3). Arginine remains predominant at the fourth position while presence of serine, glutamine, and other amino acids at this position is highly nonsignificant (Table 1).

 In HIV-1, V3 is involved in coreceptor usage [13]. This region also contains major antigenic and neutralizing epitopes in HIV-1 which are well exposed upon CD4-binding [14–17]. Residues in the V3 crown, including the GPG motif, are important for soluble gp120-CD4 complex binding to cell surface receptor CCR5 [33]. The tip motif represents a target for neutralizing antibodies [34]. Therefore, sequence variation in this motif may have an impact on virus infectivity and disease progression.

### 3.4. Consensus of V3 Loop

**Figure 4:**
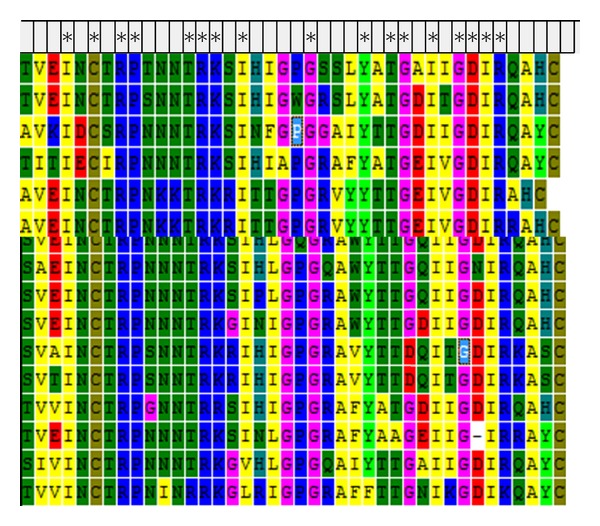
Aligned sequences exhibiting the consensus region of V3 loop (indicated by ∗).

The conserved amino acids were calculated after aligning the V3 loop sequences. Out of 35 amino acids of the V3 loop, 21 remain the same during the studied period. Hence, the 60% of amino acids of the V3 loop remain conserved (Figure 4). The conserved residues is marked (*) Figure 4.

According to previous research, some of the conserved or relatively conserved residues are normally essential for the biological functions of the HIV-1 [34]. The deletion or substitution of some residues in this loop was found to affect gp120 coreceptor interactions and may further influence the HIV-1 target entry into the host.

## 4. Conclusion

The present study is the first report that demonstrated the evolutionary pattern of HIV-1 during the period of 17 years from 1990 to 2007. The pathogenicity and infectivity of the HIV-1 subtype B is increased that showed the rapid evolutionary and modulatory mechanism. The analyzed data also provides useful information on the surveillance of HIV-1 infection in Asia and highlights the evolutionary pattern of virus populations which may be under varied selection pressures during different periods of time. However, further studies are required to get more insights of all these mechanisms and for developing effective therapies and vaccines against HIV-1 infection.

## Figures and Tables

**Table 1:** Amino acids frequency at the fourth position of the GPGX tetramer of V3 loop of HIV-1 subtype B.

Amino acid	Percentage of subtype B isolates in Asia
Arginine (R)	73%
Glutamin (Q)	11%
Serine (S)	8%
Others	8%
